# Mortality Evaluation and Life Expectancy Prediction of Patients with Hepatocellular Carcinoma with Data Mining

**DOI:** 10.3390/healthcare11060925

**Published:** 2023-03-22

**Authors:** Che-Yu Liu, Chen-Yang Cheng, Szu-Ying Yang, Jyh-Wen Chai, Wei-Hao Chen, Pi-Yi Chang

**Affiliations:** 1 Department of Radiology, Taichung Veterans General Hospital, Taichung 407, Taiwan; 2 Department of Industrial Engineering and Management, National Taipei University of Technology, Taipei 106, Taiwan; 3 Nursing Department, Taichung Veterans General Hospital, Taichung 407, Taiwan; 4 Section of Radiology, College of Medicine, China Medical University, Taichung 404, Taiwan; 5 College of Medicine, National Chung Hsing University, Taichung 402, Taiwan; 6 Institute of Business & Management, National Yang Ming Chiao Tung University, Taipei 100, Taiwan; 7 Department of Industrial Engineering and Enterprise Information, Tunghai University, Taichung 407, Taiwan

**Keywords:** hepatocellular carcinoma (HCC), decision tree, transarterial chemoembolization (TACE)

## Abstract

Background: The complexity of systemic variables and comorbidities makes it difficult to determine the best treatment for patients with hepatocellular carcinoma (HCC). It is impossible to perform a multidimensional evaluation of every patient, but the development of guidelines based on analyses of said complexities would be the next best option. Whereas conventional statistics are often inadequate for developing multivariate predictive models, data mining has proven more capable. Patients, methods and findings: Clinical profiles and treatment responses of 537 patients diagnosed with Barcelona Clinic Liver Cancer stages B and C from 2009 to 2019 were retrospectively analyzed using 4 decision tree algorithms. A combination of 19 treatments, 7 biomarkers, and 4 states of hepatitis was tested to determine which combinations would result in survival times greater than a year in duration. Just 2 of the algorithms produced complete models through single trees, which made them only the ones suitable for clinical judgement. A combination of alpha fetoprotein ≤210.5 mcg/L, glutamic oxaloacetic transaminase ≤1.13 µkat/L, and total bilirubin ≤ 0.0283 mmol/L was shown to be a good predictor of survival >1 year, and the most effective treatments for such patients were radio-frequency ablation (RFA) and transarterial chemoembolization (TACE) with radiation therapy (RT). In patients without this combination, the best treatments were RFA, TACE with RT and targeted drug therapy, and TACE with targeted drug therapy and immunotherapy. The main limitation of this study was its small sample. With a small sample size, we may have developed a less reliable model system, failing to produce any clinically important results or outcomes. Conclusion: Data mining can produce models to help clinicians predict survival time at the time of initial HCC diagnosis and then choose the most suitable treatment.

## 1. Introductory Statement

Hepatocellular carcinoma (HCC) is the 7th most prevalent cancer in the world and has the second-highest mortality rate, with an estimated 832,000 deaths worldwide in 2020, just below the level of lung cancer [1]. Even with improved treatment protocols and medication, the expected 5-year survival rate of patients with HCC is less than 20% [2]. Later-stage HCC is associated with even worse survival rates; patients diagnosed with Barcelona Clinic Liver Cancer (BCLC) stage B have a median survival time of 21.8 months, while BCLC stage C patients have a time of only 6.6 months [3]. 

The BCLC guidelines (updated in 2022) divided treatment modalities for HCC patients into two categories: curative and palliative therapy. Therapy selection is mainly based on tumor size, number of tumors, the extent of extrahepatic invasion, performance status, biochemical profile, severity of liver disease and complexity of comorbidity. The curative route—surgical resection, liver transplantation (LT), radiofrequency ablation, and microwave ablation—is specifically preserved for early-stage HCC or a solitary lesion with preserved liver function. The palliative route includes transarterial chemoembolization (TACE), hepatic artery infusion chemotherapy (HAIC), systemic chemotherapy, immunotherapy, and trans-arterial radioembolization (TARE) with Yttrium-90 and is prescribed for intermediate-to-late-stage HCC and those with extra-hepatic involvement or deteriorated and decompensated liver cirrhosis [4].

Due to the complex interactions between patient and disease factors in HCC treatment, a multidisciplinary approach is essential. Many therapeutic options for focal advanced disease have limitations in clinical practice. For example, TACE is contraindicated in patients with both intermediate HCC and untreatable arteriovenous fistula, since embolization may be expected to bring more harm than benefit. RFA is also contraindicated in patients with bleeding diathesis, even if the tumor size and location are suitable for this procedure. A successful treatment algorithm must account for all of these factors when generating its recommendations, which is difficult for the conventional statistical techniques utilized by most existing clinical studies. In recent years, novel techniques for data analysis have been developed to provide more nuanced and comprehensive clinical insights. Data mining, also known as “knowledge discovery in databases” (KDD), was applied in this study to explore the interconnections between multiple confounding variables affecting HCC treatment. 

Several data mining strategies have been proposed, such as decision trees, clustering, association rules, artificial neural networks, etc. [5,6]. The concept behind decision trees is to develop a flow-chart-like model that can be generated from a root node and then through multiple internal nodes to reach the leaf nodes. Decision trees are able to deal with both discrete and continuous variables, handle records with missing values, and subdivide heavily skewed continuous data into ranges [7,8]. Besides, decision trees are also free of ambiguity, easy to interpret and use, and able to compare or correlate with current medical concepts developed using traditional statistic techniques [5]. 

As a consequence, this study proposed a data mining model to evaluate the relationships between biochemical profiles, treatment options and survival periods for those patients with intermediate- to advanced-stage HCC (BCLC stages B and C). With the aid of the models that were output, we sought to produce biochemical cut-off levels with which we could predict the expected survival time for every BCLC stage B and C patient. 

## 2. Methods and Materials 

This retrospective study was approved by the Institutional Review Board of Taichung Veterans General Hospital (IRB No. CE17306A), which waived the requirement for informed consent. We collected the clinical profiles and data of patients diagnosed with intermediate (BCLC stage B) and advanced HCC (BCLC stage C) from 2009 to 2019 from the archives of the Informatics Research and Development Center of Taichung Veterans General Hospital. Biological profiles were collected during a period of two months—one month before and after treatment. The characteristics or information of interest included treatment type, serocondition of viral hepatitis (hepatitis B, hepatitis C, hepatitis B&C, none), and survival time. Biological profile information was also collected: glutamic oxaloacetic transaminase (GOT), also known as aspartate aminotransferase (AST); glutamic pyruvic transaminase (GPT), also known as alanine aminotransferase (ALT); albumin (ALB); international normalized ratio (INR); alpha-fetoprotein (AFP); assessment information for retreatment with TACE score (ART); and total bilirubin (T-Bil).

The treatments we collected data for were as follows: (1) surgery; (2) systemic chemotherapy; (3) TACE; (4) symptomatic treatment (including palliative treatment); (5) refused treatment; (6) radiation therapy; (7) radio frequency ablation (RFA); (8) targeted drug therapy; (9) liver transplant; (10) immunotherapy; (11) Yttrium-90; (12) surgery + targeted drug therapy; (13) TACE + radiation therapy; (14) TACE + radiation therapy + targeted drug therapy; (15) TACE + radiation therapy + targeted drug therapy + immunotherapy; (16) TACE + targeted drug therapy; (17) TACE + targeted drug therapy + immunotherapy; (18) radiation therapy + targeted drug therapy; and (19) targeted drug therapy + immunotherapy. The key considerations for each HCC treatment modality are as follows:

Systemic chemotherapy: No HCC patient received systemic cytotoxic chemotherapy in this study. This was due to the elevated risk of adverse reactions such as myelosuppression.

TACE: Depending on tumor size and patients’ blood panel results, up to 40 mg of doxorubicin may be used during TACE. At our institution, factors such as large tumor size and abnormal liver function are indications for a more cautious and selective approach to chemoembolization. Modifications to the TACE protocol such as decreasing the dose of epirubicin or foregoing Gelfoam cubes may also be implemented, especially in patients with main portal vein thrombosis.

Radiation therapy: External beam radiation therapy (EBRT) at our institution was primarily reserved for treating portal vein thrombosis involving the main trunk and/or a major branch. HCC was included in the radiation field only if the tumor was in close proximity to the thrombosis site. EBRT for patients in this study was performed for 5 days a week via a 10–15 MV linear accelerator, with a median radiation dose of 45 Gy and fraction size of 1.8–3 Gy.

Refused treatment: Four patients refused any treatment due to personal reasons.

RFA: Patients with preserved liver function, small tumor size (<3 cm), and low lesion number (≤3) were selected for RFA. Median tumor number is 1, with a median tumor size of 1.6 cm.

Targeted therapy: Three target therapy drugs were prescribed during the study period, including Sorafenib, Regorafenib, and Lenvatinib. While the exact duration of prescriptions was not well documented for each patient, the target therapy group received no other interventions.

Immunotherapy: Nivolumab was used. The exact duration of prescription was also not well documented, but this group also did not receive any other treatment modality.

Yttrium-90: A two-step procedure for Yttrium-90 therapy was adopted. First, predictive dosimetry was performed using 99mTc-MAA SPECT/CT in order to quantify the lung shunting fraction and ratio of tumor to normal tissue. The expected radiation doses to the patient’s liver, lung, and tumor were then calculated based on a partition model. A radiation dosage of up to 100 Gy may be adopted without exceeding the safety threshold for lung and normal liver tissues. After careful patient selection and pre-treatment based on results of 99mTC-MAA SPECT/CT and radiation dose modeling, radioembolization with Yttrium-90 was performed. 

Surgery: Wedge resection, segmentectomy and lobectomy with adequate surgical margins (>1 cm) were performed according to the expertise of specialist liver surgeons. At our institution, patients with multiple HCCs in one lobe were still eligible for curative lobectomy if clinical conditions permitted.

Survival time was divided into two categories: “≤1 year” and “>1 year.” The 1-year cutoff was chosen based on studies that indicate that roughly 50% of liver cancer patients survive for more than a year, which allowed for our data to be balanced. The date of initial HCC diagnosis, the date of last contact or death, and the survival status at the latter were all collected and used in this analysis.

### Data Preprocessing and Variables Conversion

To make non-numeric data usable by machine learning models, they were converted into numeric data using one-hot encoding. Numerical data, like the six categories of blood parameters, was not converted.

If there was a missing value in a column, this patient was excluded from the study, as that meant that they may not have had the test performed and therefore could not undergo the same analysis as the other patients. Since decision trees are prone to overfitting if presented with outliers in the training data, patients with extreme outliers in blood test results (e.g., AFP > 484,000 mcg/L, etc.) were also excluded to improve the accuracy of the predictive models [9,10,11,12,13].

## 3. Data Mining Model

### 3.1. CART

In this study, the CART algorithm, which can handle two types of data in the case hospital, was used to calculate the *Gini* coefficient to construct a decision tree model. At each node, the CART algorithm tried to use each feature (i.e., input variable) for classification and compared the *Gini* coefficient under each feature classification, finally selecting the feature with the smallest *Gini* coefficient as the classification method of the node. The *Gini* coefficient could be interpreted as the possibility that the sample was wrongly classified in the subset (its data impurity, so to speak). As such, the smaller the *Gini* coefficient was, the clearer the classification result of the feature was also. If a node had *D* samples in its training set and *i* categories in its target variable, *p_i_*^2^ was the square of the probability of category *i*, and its *Gini* coefficient was:

(1) $$Gini\left(D\right)=1-\overset{n}{\underset{i=0}{\sum }}p_{i}^{2}$$

The best case for the *Gini* coefficient was 0, i.e., $p_{i}=1$ and $Gini\left(D\right)=0$. In this case, all samples could be completely classified into the same category, indicating that the data impurity at this time was at its lowest. 

### 3.2. Post-Pruning CART (PPC)

To both simplify the model and improve its accuracy, post-pruning of the CART results was performed by combining nodes with similar results. Ambiguous results were also pruned; for example, a hypothetical node with a 59% probability of “survival > 1 year” and a corresponding 41% probability of “survival ≤ 1 year” would not have been easily classified as one or the other and would therefore have been pruned. 

### 3.3. Random Forest

Random forest is an extension algorithm of decision trees. Multiple decision trees were combined into a “forest,” and each decision tree in the forest was trained with data randomly allocated by the bootstrap method. Finally, bagging was used to give each tree a vote, and the result obtained by the majority vote was used as prediction. 

Given a training set *X* = *x*_1_,…, *x_n_*, and its corresponding *Y = y*_1_*,…, y_n_*, *K* random sampling *k* = 1 , 2, …, *K* from the *n* samples of the training set was repeated (i.e., *K* trees were generated). The sample extracted from training set was *X_k_, Y_k_,* and the number of the sample was *n_k_*. The features of *X_k_* were also selected by random extraction, and the decision tree model *f_k_* of the *K^th^* tree was trained by the sample from training set *X_k_, Y_k_*. Finally, the sample was put back into the whole training set, meaning that each sampling was randomly selected from *n* samples. When the *K^th^* tree was trained, the random forest model was generated:

(2) $$\hat{f}=\frac{1}{K}\overset{K}{\underset{k=1}{\sum }}f_{k}\left(x^{\prime }\right)$$

The target variable of this study was categorical data, and there were only 2 such groups, namely, “survival > 1 year” and “survival ≤ 1 year”. If the number of votes in one of the groups was more than *K*/2, then that group was selected as the prediction. To avoid a tied vote situation, K was always an odd number.

### 3.4. XGBoost

XGBoost is also an algorithm for decision tree extension. It differs from random forest in that random forest uses bagging and each tree is independent, whereas XGBoost uses Boosting and each tree is related. XGBoost collected many weak learners into a strong learner (“boosting”); i.e., before each tree was generated, XGBoost increased the error weight of the previous tree, so that the new tree would “learn” the information of the previous errors and optimize the accuracy of the model.

### 3.5. Model Evaluation

Regarding classification, accuracy estimation is mainly based on the confusion matrix (aka “classification matrix” or “contingency table”). The accuracy of the model evaluated by a confusion matrix can be used to measure the confidence level of the classification. 

There are five common evaluation indicators for classification:

$$\mathrm{True}\;\mathrm{Positive}\;\mathrm{Rate}=\frac{TP}{TP+FN}$$

Divide the number of true-positive (*TP* for short) classifications by the total number of positive classifications. True Positive Rate is also called Sensitivity.

$$\mathrm{True}\;\mathrm{Negative}\;\mathrm{Rate}=\frac{TN}{TN+FP}$$

Divide the number of true-negative (*TN* for short) classifications by the total number of negative classifications. True Negative Rate is also called Specificity.

$$\mathrm{Precision}\;=\frac{TP}{TP+FP}$$

Divide the number of *TP* classifications by the sum of the number of *TP* classifications and the number of false-positive (*FP* for short) classifications.

$$\mathrm{Recall}\;=\frac{TN}{FN+TN}$$

Divide the number of *TP* classifications by the sum of the number of *TP* classifications and the number of false-negative (*FN* for short) classifications.

$$\mathrm{Accuracy}\;=\frac{TP+TN}{TP+TN+FP+FN}$$

The number of correct classifications is divided by the total number of testing sets. 

Straus, Sharon E. et al. indicated that clinical significance can be evaluated through the likelihood ratio [14]. The likelihood ratio (*LR*) was calculated as follows:

(3) $$LR\left(+\right)=\frac{\mathrm{Sensitivity}\;}{1-\;\mathrm{Specificity}}$$

(4) $$LR\left(-\right)=\frac{1-\;\mathrm{Sensitivity}}{\mathrm{Specificity}}$$

If *LR*(+) of the model was greater than 2, and *LR*(−) was less than 0.5, the model was considered to have a significant degree of confidence. If the value of either *LR*(+) or *LR*(−) was between 0.5 and 2, the model was not considered to have clinical significance. 

#### Descriptive Statistics

After data preprocessing, a total of 537 patients (401 men, 136 women; mean patient age: 65.7 ± 11.8 years) with liver cancer from 2009 to 2019 were included in this study. The classification of performance status is based on the ECOG Scale, with scale 0: 426, scale 1: 74, scale 2: 74, scale 3: 9 and scale 4/5: 0. The majority of patients had preserved liver function, with 293, 233 and 11 patients having Child–Pugh score of A, B, and C, respectively. A total of 63 patients had metastatic disease, with the lungs being the most common site of metastasis. There were 1 target variable and 29 input variables. The data were divided into 375 samples for the training set and 162 samples for the testing set. The distribution of categorical data is shown in Table 1, and the continuous data are shown in Table 2.

In terms of categorical data, survival time was the target variable for categorical data and was divided into “>1 year” or “≤1 year”. Each patient “adopted” only 1 of the 19 afore-mentioned treatments. Hepatitis is also mentioned in the Chapter Method, where it is elaborated that each patient with advanced liver cancer will only select one of the four hepatitis types as “yes”.

## 4. Results

**Figure 1:**
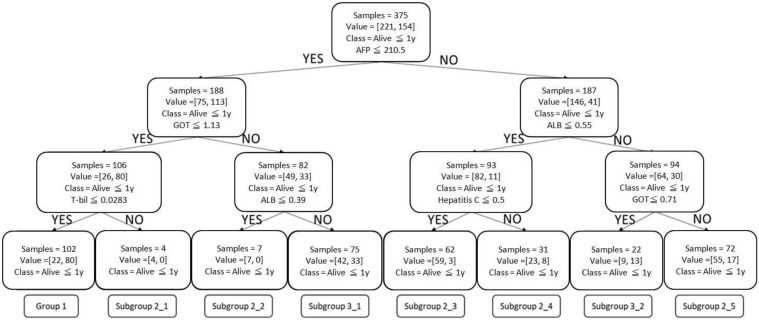
CART under Maximum 3 Layers.

**Figure 2:**
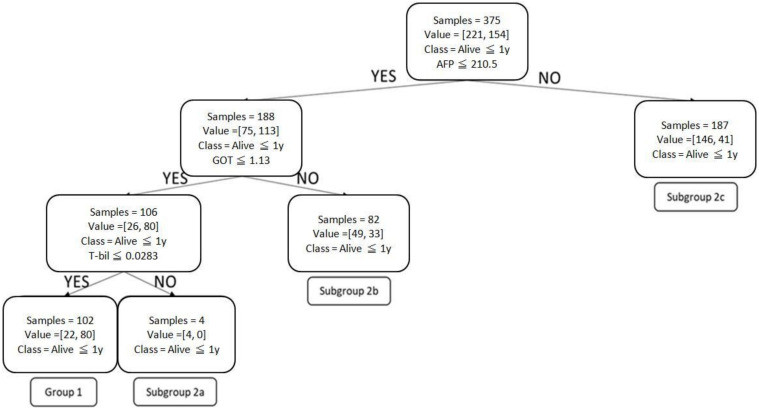
Post-pruning CART.

CART divided the patients into 3 groups, based on “survival greater than 1 year” of less than 40%, between 40% and 60%, and greater than 60% (Figure 1 and Table 3). Post-pruning CART combined such subgroups into one (Subgroup 2b in Figure 2), which creates a simpler yet accurate induction method for clinical use. 

To ensure that each model had a reasonable level of accuracy and feasibility, this study used a confusion matrix to calculate the sensitivity and specificity of each model. The use of different models specifying the maximum number of layers resulted in different accuracy scores. After selecting the models with the highest accuracy scores and specifying the maximum number of layers, *LR*(+) and *LR*(−) were then used to determine whether the models had clinical significance. Credible models were those with an *LR*(+) greater than 2 and *LR*(−) less than 0.5; any scores were not considered clinically significant. 

All four models were considered credible and therefore clinically significant (Table 4). The random forest method had the highest level of accuracy; however, its output was not as easy to read as those of CART or post-pruning CART, and physicians would have needed to perform computer programming within the clinic to obtain usable prediction results. Therefore, this study chose to focus on post-pruning CART, which had the second highest accuracy, along with CART. As mentioned above, post-pruning CART had the further benefit of grouping together subgroups with the same result.

Table 5 breaks down the most commonly seen treatments for the patients in each subgroup of post-pruning CART, which were Group 1 and Subgroups 2a, 2b, and 2c, and the treatments with the highest >1-year survival rates. For example, in Group 1′s 375-sample training set, 102 patients showed a combination of alpha fetoprotein ≤210.5 mcg/L, glutamic oxaloacetic transaminase ≤1.13 µkat/L, and total bilirubin ≤ 0.0283 mmol/L, of which 80 patients survived for more than 1 year (>1-year survival rate of 78%). Surgery was the most commonly seen Group 1 treatment—45 patients underwent a procedure, accounting for 44% of the cohort. RFA was the most effective Group 1 treatment; a total of 7 patients received this treatment and survived for more than 1 year (>1 survival rate of 100%). This was followed by TACE + radiation therapy, received by 1 patient, which also had a 100% > 1 year survival rate.

The most commonly adopted treatments were surgery in Group 1 and TACE, symptomatic treatment (including palliative treatment), radiation therapy, and immunotherapy in the three Group 2 subgroups. The best treatments, in terms of >1-year survival rates, were RFA in Group 1 and RFA, TACE + radiation therapy + targeted drug therapy in the second group, and TACE + targeted drug therapy + immunotherapy in Group 2 subgroups.

## 5. Discussion

In this study of 537 patients with BCLC stage B or C HCC, we used four different data mining algorithms to seek out predictive relationships between survival rate and various biochemical markers that would be known at the time of HCC diagnosis. We set the survival interval cutoff value to be 1 year, which was in accordance with prior studies. According to previous study, the 1-year survival rate of 1 year for BCLC B and BCLC C was 52.5% and 27.0%, respectively. Additionally, in the study, which included total 1637 patients diagnosed with HCC of BCLC B and BCLC C, the 1 year survival rate was 41.5% [15]. Wang et al.’s study included 2347 patients with BCLC B and C disease and showed a 1-year survival rate 38.7% [16]. Another meta-analysis showed that the pooled BCLC B+C disease have 1-year survival rate of 34% (95% CI, 22–48) [17]. Other research has shown an overall survival time for HCC patients to be less than 20 months, a prediction which even includes the earliest stages of HCC [1].

Our model was developed to predict expected survival times based on the biochemical profile and treatment. The CART model with three layers presented a positive prediction rate of 72.2%. The first layer of CART was AFP, with a cutoff value of 201.5(mcg/L). In a prospective study, Gomez-Rodriguez et al. analyzed the relationship of BCLC and blood concentration of AFP to prognosis in 136 patients, subdividing serum AFP levels into three groups: AFP ≤ 20, 20–200 and >200(mcg/L). The median survival among each group was: AFP ≤ 20, 62.27 months; 20–200, 22.08 months; and >200 mcg/L, 5.39 months, which was considered significant (*p* < 0.0001) [18]. A retrospective study found that HCC patients with high AFP tended to have more aggressive presentations of HCC, including greater tumor burden, massive or diffuse types, and portal vein thrombosis, which made AFP level a negative prognostic indicator [19]. 

HCC patients in this study were treated based on both BCLC and consensus guidelines by the Taiwan Liver Cancer Association. If extrahepatic spread had already been present upon diagnosis, systemic therapy would be favored. In such cases, the patient’s liver function, intrahepatic tumor condition and the extent of vascular or portal invasion would be the focus of pre-treatment planning. In contrast, resection and local ablation techniques such as RFA have a larger role to play in the treatment of early HCC. Interestingly, while BCLC generally recommends surgical resection only in early HCC, indications for partial hepatectomy have been expanded according to consensus guidelines in Taiwan. Accordingly, curative resection may even be performed at our institution in patients with hepatic vein thrombosis ipsilateral to the tumor. Factors that make this possible include advances in surgical techniques, intensive post-operative care and careful case selection based on comprehensive evaluation of patient condition and liver function [20,21,22,23]. Regardless, TACE becomes a more promising treatment modality as tumor size and lesion number increases, making complete resection or local ablation less feasible.

TACE is one of the leading treatment options to prolong the survival of patients with intermediate to advanced HCC. However, of major concern to clinicians is post-TACE hepatic failure, for which no concrete or standardized definition currently exists, which makes comparing datasets from different studies or institutes difficult due to the heterogeneity of definition. Previous studies have reported a wide range of acute hepatic failure rates (0–48.6%), averaging 7.5% of patients, with most functional deteriorations restored to pre-TACE level before the next treatment episode and only 3.0–5.7% of patients suffering from irreversible functional damage [24,25]. Higher baseline serum total bilirubin has been found to be related to the occurrence of hepatic failure after TACE; however, the level of significance is inhomogeneous, which may be due to inconsistent use of definitions in these studies [24,25,26,27,28,29]. Two studies—one with 403 patients enrolled and the other with 268—revealed T-Bil to be a statistically significant independent predictor of survival in both univariate and multivariate analysis [26,30]. Tateishi et al. divided T-Bil levels into three groups—< 0.017, 0.017–0.034, and >0.034 mmol/L—and found that only the latter two were significantly correlated with survival rate (*p* = 0.0016 and < 0.0001, respectively). Greico et al. divided T-Bil level into two groups—<0.0257, and >0.0257 mmol/L—and found that the latter had a significant negative correlation with survival. In our study, T-Bil was one variable in the second layer of CART, with a cutoff value of 0.0283 mmol/L, which is in line with the conclusions of both Tateishi and Greico. Interestingly, Huang et al. found the monoethylglycinexylidide (MEGX) test to be a superior predictor of hepatic failure than T-Bil, with good sensitivity and specificity (94.7% and 97.6%, respectively), which presents a possible further area of study [29].

Recent studies have pointed out that albumin may play a role in growth inhibition and may have the ability to suppress HCC proliferation by seizing the cell cycle of HCC in the G1 phase [31,32]. Albumin levels are known to be closely related to liver function and this level is one of five characteristics that make up the Child–Pugh score. In a univariate analysis of 268 untreated patients, Grieco et al. found that albumin levels of <0.53 mmol/L had a significant negative correlation with survival time—22.3 months, versus 29.6 for ≥0.53 mmol/L (*p* < 0.001) [30]. Tateishi et al. found albumin levels of <0.53 mmol/L to be a significant independent predictor of survival in both univariate and multivariate analysis [26]. Our CART model produced two cutoff values, 0.39 and 0.55 mmol/L.

In addition, we also found that when patients with hepatitis and similar blood test results were compared, those with hepatitis B were prone to worse outcomes. Choi et al. found testing either hepatitis B- or hepatitis C-seropositive would worsen the patients’ survival time, with the former having a more significant impact [33]. However, both our results and Choi’s are exceptions to what has been shown elsewhere. Other studies have concluded that HCC patients with hepatitis C presented significantly higher hazard ratios in survival time [34,35]. Similarly, other research has shown that hepatitis C has a larger impact on life expectancy than hepatitis B, regardless of HCC diagnosis [36,37]. The incongruities between these findings and ours may be due to the differences between the computational methods of data mining and those of traditional statistics.

When analyzing Group 1, we found that, although surgery was the most commonly used therapy for BCLC stages B and C of HCC, RFA provided superior outcomes. However, prior research has found that RFA has significantly poor overall survival rates and recurrence-free survival in the following 5 years [38,39,40,41]. Nevertheless, RFA could be deployed as part of a combination therapy to optimize the efficacy of treatment for patient with poor hepatic function or unresectable HCC. RFA is also a choice for bridging therapy before liver transplantation, with some studies finding that bridging with RFA could decrease the dropout rate approximately 10–20%. The superior outcome of RFA in our study may have been due to a small sample size, some unseen selection bias, or those particular patients not being suitable for surgery because of physical status or anesthetic risk.

In our prediction model, most of the cutoff levels of the biochemical indicators were comparable to those found in the literature. Although our sample size was small for data mining in other fields, such as financial or marketing, our model was still able to produce results similar to those of literature based on classical statistics. However, our model also produced some contrary results; for example, in our model, patients with hepatitis and similar blood test results largely had hepatitis C and were likely to survive for more than 1 year, which conflicts with the results of most other studies. This led us to speculate that this sample size may have been too small to evoke the full potential of data mining, causing some concern about the accuracy of the model. 

This study had a number of limitations. First of all, as a retrospective study, selection bias was inevitable, which made the enrolled dataset less representative of the target group, which comprised patients with HCC in BCLC stages B and C. Second, the sample size and training set were both relatively small, which may have caused the development of a less reliable model system and may have failed to produce any clinically important results or outcomes. In spite of these shortcoming, this pilot study has allowed us to develop and test new methods and techniques for predictive modeling. Once more patients with detailed clinical parameters have been enrolled to fine-tune the QSP model, it can be used to generate virtual patients that mitigate gaps in available clinical data [42]. With a larger sample size, not only would have the study sample more closely represented the actual population, but more datasets could have been used to train the model system, creating more reliable model systems. A third limitation is that several subgroups in the training set were composed of a limited number of patients, especially for Yttrium-90 and combination treatment. As a relatively novel treatment technique in Taiwan, Yttrium-90 therapy for HCC has only been available at our institution since the end of 2019. As for combination treatments, many are based on multidisciplinary approaches and required extensive discussions between specialists in different fields. Since most of the resulting treatment regimens are highly individualized, only a small number of patients received them. Since these patients still fit the study’s inclusion criteria, we did not think it was appropriate to exclude them from model training. Fourth the survival status and exact survival period were difficult to identify from the patient data culled from the Informatics Research and Development Center of our institute. If more precise survival data had been available to use to train the model system, we believe the ability of survival prediction would have improved dramatically. In the future, it may be possible to provide predictions with narrower intervals, rather than just with a dichotomized variable greater than or less than 1 year as cutoff value, which, while based on other studies in the literature, was admittedly somewhat arbitrary. Fifth, while this study focuses on applying data mining to biological profiles to suggest appropriate therapy strategies, in reality it is impossible to treat HCC patients without the use of imaging studies such as CT or MRI. Therefore, it will be necessary to integrate relevant image findings into future prediction models. Lastly, the biochemical profiles analyzed in this study were somewhat lacking in fine details. Subdividing the dataset using more sophisticated and in-depth characteristics would improve the reliability and strength of our prediction model.

In conclusion, this study developed a model that could help clinicians to predict patient’s survival time based only on the biochemical profile data available at the initial diagnosis of HCC and then choose the most suitable treatment based on objective data instead of relying solely on personal clinical experience. The results and cutoff values were largely comparable to those from other studies that were based on classical statistics, except for the viral etiology of hepatitis as a prognostic indicator. To develop improved models, larger data sets with more nuanced data must be used. 

## Figures and Tables

**Table 1:** The Distribution of Categorical Data.

Variables		Categories	Training Set (*n* = 375)	Testing Set (*n* = 162)	Total Set (*n* = 537)
Survival Time		>1 year	154	71	225
		≤1 year	221	91	312
Treatment					
1	Surgery	adopt	105	47	152
2	Systemic chemotherapy	adopt	0	0	0
3	TACE	adopt	156	69	225
4	Symptomatic treatment (including palliative treatment)	adopt	25	10	35
5	Refused treatment	adopt	3	1	4
6	Radiation therapy	adopt	14	4	18
7	RFA	adopt	9	2	11
8	Targeted drug therapy	adopt	36	18	54
9	Liver transplant	adopt	0	0	0
10	Immunotherapy	adopt	1	0	1
11	Yttrium 90	adopt	0	0	0
12	Surgery + targeted drug therapy	adopt	1	0	1
13	TACE + radiation therapy	adopt	2	0	2
14	TACE + radiation therapy + targeted drug therapy	adopt	5	3	8
15	TACE + radiation therapy + targeted drug therapy + immunotherapy	adopt	0	0	0
16	TACE + targeted drug therapy	adopt	12	5	17
17	TACE + targeted drug therapy + immunotherapy	adopt	1	1	2
18	Radiation therapy + targeted drug therapy	adopt	3	2	5
19	Targeted drug therapy + immunotherapy	adopt	2	0	2
Hepatitis 1: Non-Hepatitis		yes	81	28	109
Hepatitis 2: Hepatitis B		yes	164	69	233
Hepatitis 3: Hepatitis C		yes	114	58	172
Hepatitis 4: Hepatitis B and C		yes	16	7	23

Abbreviation: TACE, transarterial chemoembolization; RFA, radiofrequency ablation.

**Table 2:** The Distribution of Continuous Data.

Variables	Training Set (*n* = 375), Avg	Testing Set (*n* = 162), Avg	Total Set (*n* = 537), Avg
GOT (µkat/L)	1.51	1.62	1.54
GPT (µkat/L)	1.28	1.23	1.26
ALB (mmol/L)	0.55	0.55	0.55
INR	1.11	1.10	1.10
T-bil (mmol/L)	0.02	0.02	0.02
AFP (mcg/L)	21,003.15	19,673.33	20,601.97

Abbreviation: GOT, glutamic oxaloacetic transaminase (GOT); GPT, glutamic pyruvic transaminase; ALb, albumin; INR, international normalized ratio; T-Bil, total bilirubin; AFP, alpha-fetoprotein (AFP).

**Table 3:** The Classification of CART.

					Input Variables				
Group	Subgroup	*n* = 375	1-Year Survival	Prediction	AFP (mcg/L)	GOT (µkat/L)	ALB (mmol/L)	T-Bil (mmol/L)	Hepatitis C
1		102	78%	>1 year	≤210.5	≤1.13	-	≤0.0283	-
2	2-1	4	0%		≤210.5	≤1.13	-	>0.0283	-
	2-2	7	0%		≤210.5	>1.13	≤0.39	-	-
	2-3	62	5%	≤1 year	>210.5	-	≤0.55	-	No
	2-4	31	26%		>210.5	-	≤0.55	-	Yes
	2-5	72	24%		>210.5	>0.71	>0.55	-	-
3	3-1	75	44%	≤1 year	≤210.5	>1.13	>0.39	-	-
	3-2	22	59%	>1 year	>210.5	≤0.71	>0.55	-	-

**Table 4:** Accuracy of all Models.

	Accuracy	Sensitivity	Specificity	*LR*(+)	*LR*(−)
CART	0.72	0.70	0.77	3	0.39
Post-pruning CART	0.72	0.90	0.48	2	0.21
Random forest	0.76	0.74	0.80	4	0.33
XGBoost	0.70	0.71	0.69	2	0.42

**Table 5:** Clinical Use of Post-pruning CART.

					Input Variables			Most Common Treatment(s) No. of Patients (1-Year SR, %)	Treatment with Highest SR No. of Patients (1-Year SR, %)	Treatment with 2nd-Highest SR No. of Patients (1-Year SR, %)	Treatment with 3rd-Highest SR No. of Patients (1-Year SR, %)
Prediction	Group	Subgroup	*n* = 375	1-year Survival	AFP (mcg/L)	GOT (µkat/L)	T-bil (mmol/L)				
SR > 1 year	1		102	78%	≤210.5	≤1.13	≤0.0283	Surgery 45 patients (44%)	RFA 7 patients (100%)	TACE + RT 1 patient (100%)	Surgery 45 patients (82%)
SR ≤ 1 year	2	2a	4	0%	≤210.5	≤1.13	>0.0283	TACE 1 patient (25%) ST 1 patient (25%) RT 1 patient (25%) IT 1 patient (25%)			
		2b	82	40%	≤210.5	>1.13		TACE 41 patients (50%)	RFA 1 patient (100%) TACE + RT + TDT 1 patient (100%)	Surgery 20 patients (50%) RT 2 patients (50%)	TACE 41 patients (41%)
		2c	187	22%	>210.5			TACE 75 patients (40%)	RFA 1 patient (100%) TACE + TDT + IT 1 patient (100%)	TACE + TDT 5 patients (60%)	RT + TDT 2 patients (50%)

SR—survival rate; No.—Number of patients; RFA—radiofrequency ablation; TACE—transarterial chemoembolization; RT—radiation therapy; ST—symptomatic treatment (including palliative treatment); TDT—targeted drug therapy; IT—immunotherapy.

## Data Availability

The data are not publicly available due to privacy restriction.
